# Microbiological and Clinical Evaluation of the Efficacy of a Chemical Desiccant Agent in Non-Surgical Periodontal Treatment: A Randomized Controlled Clinical Trial

**DOI:** 10.3390/antibiotics14101050

**Published:** 2025-10-20

**Authors:** Alessia Pardo, Gabriele Brancato, Annarita Signoriello, Elena Messina, Giovanni Corrocher, Valentina Bellopede, Gloria Burlacchini, Caterina Signoretto, Giorgio Lombardo

**Affiliations:** 1 Dentistry and Maxillofacial Surgery Unit, Department of Surgery, Dentistry, Pediatrics and Gynecology (DIPSCOMI), University of Verona, Piazzale L.A. Scuro, 10, 37134 Verona, Italy; alessia.pardo@univr.it (A.P.); gabriele.brancato.98@gmail.com (G.B.); giovanni.corrocher@univr.it (G.C.); giorgio.lombardo@univr.it (G.L.); 2 G.B Rossi, Integrated University Hospital of Verona, Piazzale L.A. Scuro, 37134 Verona, Italy; valentina.bellopede@aovr.veneto.it; 3 Diagnostic and Public Health Department, University of Verona, 37134 Verona, Italy; gloria.burlacchini@univr.it (G.B.); caterina.signoretto@univr.it (C.S.)

**Keywords:** desiccant agent, periodontopathogens bacteria, non-surgical therapy, randomized clinical trial

## Abstract

**Background:** This randomized clinical trial compared the effects of topical irrigation with a desiccant agent (HybenX Oral Tissue Decontaminant, HBX) combined with full-mouth ultrasonic debridement as well as scaling and root planing (FMUD-SRP) versus conventional non-surgical periodontal therapy (US-SRP). **Methods**: Three quadrants per patient with probing pocket depth (PPD) ≥ 5 mm were randomly assigned to (i) the control group (US-SRP only), (ii) test group 1 (HBX + US-SRP at baseline, HBX 1T (one time)), or (ii) test group 2 (HBX + US-SRP across three sessions, HBX 3T (three times)). Clinical parameters included probing pocket depth (PPD), bleeding on probing (BOP), plaque index (PI), gingival recession (REC), and Clinical Attachment Level (CAL), recorded at baseline (Tbase), 45 days (T45d), and 90 days (T90d). Microbiological sampling was conducted for all sites at Tbase, T45d, and T90d to assess periodontal pathogens. HBX-treated sites received gel application for 60 s, followed by a saline rinse and US-SRP. **Results**: Significant differences were found between groups in PPD (*p* = 0.04) and CAL (*p* = 0.02) at T45d versus Tbase, while BOP, PI, and REC showed no significant inter-group differences at T45d. The HBX 3T group demonstrated greater pathogen reduction compared to the control and HBX 1T groups, except for one bacterial species. **Conclusions**: All treatments improved clinical and microbiological parameters. Even if single and triple applications of HBX showed similar clinical results, the repeated application achieved greater bacterial reduction.

## 1. Introduction

Periodontal disease is widespread both in developed and developing countries, affecting approximately 20–50% of the global population. Its high prevalence among adolescents, adults, and elderly individuals makes it a significant public health concern [1]. Chronic periodontitis (CP) [2] is an inflammatory disease caused by oral biofilm that often leads to the destruction of the tooth-supporting structures and, ultimately, tooth loss. Periodontitis is defined as the presence of gingival inflammation in sites with pathological detachment of collagen fibers from the cementum and apical migration of the junctional epithelium. The inflammatory events associated with connective tissue attachment loss finally result in alveolar bone resorption [3].

Current evidence supports that not all individuals have the same susceptibility to develop periodontitis. As a combination of environmental and genetic factors influences the host response to microbial plaque, the current literature [4,5] reports techniques of periodontal treatment by the elimination of biofilm to a level that allows for adequate host response control. In this proposal, non-surgical periodontal therapy, both mechanical and manual, is considered the gold standard [6]: its success primarily depends on the effective removal of supragingival and subgingival bacterial biofilms containing pathogens and endotoxins. However, mechanical therapy may fail to eliminate pathogenic species due to limited access to root surfaces and to the invasive properties of specific periodontal bacteria [7], with limited effectiveness in deep periodontal pockets [8].

Microorganisms of the biofilm are embedded in a matrix [9] which prevents antimicrobial agents from effectively reaching bacterial targets in both supragingival and subgingival areas. Therefore, procedures aimed at eradicating periodontal pathogens are considered of great interest, especially regarding the potential use of antibiotics or antiseptics in this context [10]. Several studies assessed the adjunctive role of chemical and antimicrobial agents in facilitating the mechanical removal of microbial oral biofilm from tissue surfaces during scaling and root planing (SRP) procedures [11].

For many years, desiccant agents have been used in dentistry, particularly in the treatment of aphthous stomatitis [9]. Subsequently, a new generation of dental plaque desiccants was synthesized to replace the older formulations. The current product, known as HybenX gel (HBX) [12], is a liquid solution containing a concentrated mixture of sulfonic/sulfuric acids. It is employed as an adjunctive treatment not only for periodontitis, but also for peri-implantitis, peri-implant mucositis, ulcer treatment, stomatitis, and to complement endodontic therapy.

HBX exhibits antimicrobial activity against periodontal pathogen bacteria, particularly those belonging to the red and orange complexes of Socransky, and reduces inflammatory mediators associated with periodontitis [13], as demonstrated in various clinical studies and case reports. However, effective clinical efficacy for the treatment of periodontitis and data on its biocompatibility remain limited [14,15].

In addition to its antimicrobial properties, HBX represents a distinct adjunctive therapy used not only for its antiseptic effect but also as a biofilm-disrupting agent, applied prior to mechanical debridement. This application utilizes HBX’s high affinity for water to effectively absorb the aqueous component of the biofilm, promoting its denaturation and subsequent disintegration; a crucial process, given the extensively documented resilience of mature biofilms against both mechanical disruption and chemical eradication [16,17].

HYBENX has been commercially available for several years and is described by the manufacturer as a contact desiccant that facilitates biofilm removal. However, high-quality randomized evidence comparing HYBENX to other adjunctive therapies remains limited. The rationale for testing HYBENX in this study is based on its proposed biofilm-disrupting properties and smaller clinical reports, but definitive conclusions about comparative effectiveness cannot be drawn from the present trial.

Based on these considerations, authors hypothesize that this desiccant agent could represent an essential support for non-surgical periodontal therapy in reducing bacterial load and decreasing inflammation in a relatively short time, with considerable advantages in terms of management of patients’ appointments. The aim of this study was thus to evaluate the effectiveness of HybenX gel as an adjunct to periodontal debridement in patients with a diagnosis of stage II or III periodontitis (grades A to C), in comparison with conventional therapy with US-SRP.

## 2. Materials and Methods

### 2.1. Trial Design and Setting

The experimental protocol was reviewed and approved by the Ethics Committee of the University of Verona (Prot. HX-GL-ITA1; Approval Date: 20 December 2011). The study was registered on ClinicalTrials.gov (Identifier: NCT03858959) and conducted in accordance with the CONSORT guidelines for randomized controlled clinical trials [18]. This study was designed as a 10-month, split-mouth, randomized, prospective, controlled, double-blinded trial to compare the clinical and microbiological outcomes of full-mouth ultrasonic debridement (FMUD) combined with HBX application in periodontal pockets versus US-SRP alone. A secondary aim of the study was to evaluate and compare the improvement in clinical and microbiological parameters between a single application of HBX in conjunction with UD-SRP (HBX 1T, one time) and three applications of HBX with US-SRP (HBX 3T, three times). The study was conducted at the Periodontology Unit, U.O.C. of Dentistry and Maxillofacial Surgery Clinic, University of Verona, G.B. Rossi Hospital, Italy. All patients were informed about the nature of the proposed treatment and signed a written informed consent prior to participation. After signing the consent, patients were randomly assigned to control or test groups.

### 2.2. Primary Objectives

The first objective is to evaluate and compare the impact of HBX on periodontal pathogens using qualitative PCR (qPCR), specifically in terms of the presence or absence of different bacteria before and after treatment.

The second is to assess and compare the clinical effects of HBX on parameters of bleeding on probing (BOP), plaque index (PI), probing pocket depth (PPD), and Clinical Attachment Level (CAL). This randomized split-mouth trial was conducted as an exploratory study with a limited sample size; results should therefore be interpreted as preliminary. The study was not intended to provide definitive estimates of treatment effect; adequately powered randomized trials are required to confirm these findings.

### 2.3. Study Population

The study included 40 patients (15 men and 25 women), aged between 39 and 74 years (mean age 53.3 years). All participants were selected and treated from the pool of periodontal patients at the Clinic of Dentistry and Maxillofacial Surgery, University of Verona, Italy. Patient recruitment and treatment were conducted between November 2023 and March 2024.

#### 2.3.1. Eligibility Criteria

Patients aged 18 years or older were recruited from an operator involved in the clinical trial. Patients were eligible if they had a diagnosis of periodontitis within the new classification of periodontal and peri-implant diseases and if they had not undergone any other non-surgical periodontal treatment in the year preceding enrolment. Diagnostic criteria outlined in the European Federation of Periodontology (EFP) guidelines were used [19].

#### 2.3.2. Inclusion and Exclusion Criteria

Inclusion criteria were as follows:

- diagnosis of periodontitis, stage II–III, grade A/B/C, generalized form;
- at least 4 teeth per quadrant (excluding third molars);
- at least 8 teeth with pocket depth (PPD) ≥ 5 mm and radiographic evidence of bone loss; among these, at least 3 teeth with PPD ≥ 5 mm;
- male and female patients aged ≥ 18 years;
- general systemic good health as determined by medical history and clinical evaluation (ASA status I and II).

Exclusion Criteria were as follows:

- Allergy to sulfonated compounds;
- Presence of decompensated systemic diseases that could compromise study outcomes or patient safety (ASA status III and IV);
- Regular use of antibiotics;
- Regular use of anti-inflammatory drugs (NSAIDs, corticosteroids, aspirin);
- Use of anticoagulant medications;
- Severe cognitive or psychiatric disorders;
- Systemic antibiotic therapy within 1 month prior to enrolment;
- Periodontal therapy within 6 months prior to enrolment.

### 2.4. Periodontal Treatment

At the screening visit, held one week before treatment, patients completed and signed a general health and medication history form, with a full-mouth periodontal examination: sites with periodontal pockets > 5 mm in three different quadrants were randomly selected by healthcare professionals not involved in any subsequent study procedures. At the same time, patients received instructions on proper oral hygiene to improve their periodontal health and were educated on the causes and consequences of periodontal disease.

All patients received personalized home oral hygiene instructions, tailored to their dexterity, preferences, and compliance. These instructions comprised the following components: Brushing Technique: Using a manual toothbrush with either the modified Bass or Roll technique, a rotating-oscillating electric toothbrush, or a sonic electric toothbrush. Appropriate Interdental Devices: Interdental brushes or dental floss. Selection of the most suitable toothpaste for the patient. Toothpastes and/or mouthwashes containing disinfectant agents, such as chlorhexidine, were neither recommended nor prescribed. Initially, all oral hygiene techniques were demonstrated using a model or study mouth, employing the tell–show–do technique. Subsequently, the devices were applied directly in the patient’s mouth to reinforce the new home oral hygiene habit.

After periodontal evaluation at Tbase (T0), all patients received a full-mouth scaling and root planing treatment: in the test groups (HBX 1T and HBX 3T), the gel was applied topically before the US-SRP procedure. The gel was administered using a syringe with a blunt needle inserted to the bottom of the periodontal pocket and dispensed up to the gingival margin, left in place for 45–60 s, then thoroughly rinsed and aspirated using a sterile syringe with physiological saline solution. Subsequently, at Tbase, T7d (T1) and T14d (T2), patients underwent ultrasonic debridement with sharpened Gracey curettes and ultrasonic instruments (EMS Minimaster, Nyon, Switzerland). After the treatment session, all patients received instructions for at-home post-operative care. They were advised not to use antiplaque or anti-inflammatory mouthwashes or take antibiotic therapy following the procedure.

In the HBX 1T group, the gel was applied only during the first treatment session. In the HBX 3T group, the gel was applied in three sessions, with a one-week interval.

**Scheme 1:**
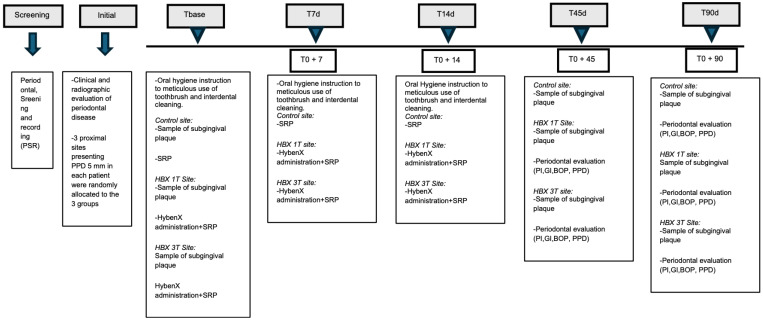
Study flow-chart. Abbreviations: d: day, SRP: scaling and root planing, PI: plaque index, GI: gingival index, BOP: bleeding on probing, PPD: probing pocket depth. Tbase: T0; T7d: T1; T14d: T2; T45d: T3; T90d: T4.

Microbiological samples of dental plaque were collected at Tbase, following the diagnosis of periodontitis, and subsequently at T45d (T3) and T90d (T4). Bacterial sampling was carried out in three periodontal pockets per patient, each with a probing depth > 5 mm and a positive bleeding on probing (BOP) response, and without endodontic involvement or furcation. Scheme 1 reports the study flow-chart.

### 2.5. Harms

Adverse events were assessed clinically and analytically at each appointment. All participants were required to report any local and systemic adverse reactions and adverse events after the topical administration using the trial’s mobile application. The severity of adverse reactions was defined using the “HOTD” guidance for oral tissue decontamination [20].

### 2.6. Sample Size

Since all the studies available in the literature present a split-mouth design, a recent one with a 6-month follow-up was chosen, assuming PPD as the primary response variable [21]. For the 6-month data, the mean difference was 0.77; the standard deviation of the differences was calculated using a previous study from the same institute as a reference, which obtained a correlation coefficient of 0.88 from the original data, yielding a standard deviation of the difference of 0.36 for this study [22]. With α = 0.05 and power 0.80, 5 pairs with a paired *t*-test were obtained. Since this was an RCT and not a split-mouth study, and since data with this study design were not available to calculate the sample size, we empirically hypothesized a study among patients with a sample of 10 patients per group (double the number).

### 2.7. Randomisation

For each patient, a computer-generated random sequence was used to assign periodontal sites (in different quadrants) to one of the following options:

- HBX application only in the first session + UD-SRP (HBX 1T);
- Repeated HybenX application over three sessions + UD-SRP (HBX 3T);
- SRP only (control group).

As previously described [23], the random list was generated using a computer software (Microsoft Excel) and defined by numbers in the ranges 0 to 50, 51 to 100, and 101 to 150, corresponding to the three groups (HBX 1T, HBX 3T, and SRP only). The sequence generation and proper allocation concealment were monitored by a dentist not involved in the participants’ enrolment.

### 2.8. Blinding

To further minimize potential bias, the study was double-blinded.

The following operators were blinded to the groups’ allocation: (i) the clinician responsible for outcome measurements during follow-up visits; (ii) the technician conducting the microbiological survey; and (iii) the statistician analyzing the data, with no access to group information. Patients were also blinded to the interventions.

Specifically [23], opaque and sealed envelopes, each containing the secret code and bearing on the outside only a number, were opened after patients’ recruitment and informed consent signing so that the investigator involved in the enrolment and treatment could not know in advance which of the 3 treatments to be performed was allocated.

### 2.9. Outcomes

Clinical parameters were recorded using a North Carolina periodontal probe (UNC-15, Hu-Friedy, Chicago, IL, USA) on all teeth at six sites per tooth: mesiolingual, lingual, distolingual, distobuccal, buccal, and mesiobuccal.

Measurements were taken at Tbase, T45d, and T90d and compared to assess clinical outcomes.

The clinical parameters evaluated were as follows:

- Bleeding on probing (BOP): This parameter was used to assess gingival inflammation. A periodontal probe was gently inserted into the gingival sulcus, and if bleeding occurred within 10–15 s, the site was considered positive. If no bleeding occurred, it was considered negative. The absence of BOP during maintenance visits has been suggested as a negative predictor for clinical attachment loss [24].
- Probing pocket depth (PPD): Measured from the gingival margin to the base of the periodontal pocket [25].
- Plaque index (PI): Measured with the probe passed along the gingival margin. PI scores ranged from 0 to 3 (0 = no plaque in the gingival area; 1 = no visible plaque, but plaque is detected on the probe tip; 2 = gingival area visibly covered with plaque; 3 = heavy plaque accumulation or calculus in the gingival area [26].
- Gingival recession (REC): Measured as the distance between the gingival margin and the cementoenamel junction (CEJ) [27].
- Clinical Attachment Level (CAL): Measured as the distance from the CEJ to the base of the sulcus or periodontal pocket [28].

One examiner not involved in the treatments recorded that all the parameters were calibrated for adequate intra- and inter-examiner levels of reproducibility before the start of the study. The calibration for intra-examiner reproducibility was performed with double recording of 10 pockets for PPD, REC, and CAL in mm, and for BOP and PI in related scores, with an interval of 24 h between the first and second recording. Basic parameters of PPD (in mm) and PI (in score) were measured for 4 pockets utilized for this purpose: average values lower than 0.1 mm for PPD and 0.5 for PI were considered reliable as threshold limits from a clinical point of view. For inter-examiner reproducibility, the above-mentioned exercise was repeated by another dentist not involved in the treatments, according to the same method.

### 2.10. Microbiological Analysis

Microbiological analysis and molecular biology investigations were conducted by the Microbiology section of the Department of Diagnostics and Public Health of the University of Verona. The analysis involved the collection and evaluation of subgingival plaque samples to detect the presence of the following periodontal pathogens:

- *Aggregatibacter actinomycetemcomitans* (Aa);
- *Porphyromonas gingivalis* (Pg);
- *Prevotella intermedia* (Pi);
- *Tannerella forsythia* (Tf);
- *Treponema denticola* (Td);
- *Actinomyces naeslundii* (An).

Plaque samples were collected from periodontal pockets with PPD > 5 mm using sterile paper points and then inserted into the appropriate medium to preserve bacterial DNA.

Microbiological sampling was performed at Tbase, T45d, and T90d.

The dental plaque sample was used to detect, by multiplex PCR (mPCR), the presence of DNA from the above-mentioned pathogens. Genomic bacterial DNA was extracted using GenElute™ Bacterial Genomic DNA Kit (Sigma-Aldrich, St. Louis, MO, USA), according to the manufacturer’s instructions, and the extracted DNA was stored at −20 °C until use. Two different multiplex PCRs (mPCR) were performed to identify the presence of *P. gingivalis*, *P. intermedia*, and *A. actinomycetemcomitans* (mPCR-M1), as well as *T. forsythia*, *T. denticola*, and *A. naeslundii* (mPCR-M2). The first multiplex PCR (mPCR-M1) used a universal 16s rRNA forward primer and a species-specific reverse primer [20]. In the second multiplex PCR (mPCR-M2), specific primer pairs for each pathogen were used [20,29].

This molecular investigation allowed us to compare the microbiological efficacy with standard UD-SRP therapy versus the adjunctive use of HBX with US-SRP, as well as to compare single and triple applications of the gel in terms of bacterial reduction [23,30].

### 2.11. Statistical Analysis

The database was created using Microsoft Excel 365, and appropriate data validation checks were performed to eliminate entry errors. Data analysis was conducted using Stata v.13.0 for Macintosh (StataCorp, College Station, TX, USA).

Descriptive statistics were used to summarize demographic and clinical characteristics. For continuous variables with a Gaussian distribution, mean and standard deviation (SD) were reported. For non-Gaussian continuous variables, median and interquartile range (IQR) were used. Categorical variables were reported as frequency distributions.

The Shapiro–Wilk test was used to assess normality. Associations between qualitative variables were analyzed using Pearson’s Chi-square test or Fisher’s exact test (when expected cell counts were <5). Comparison between means of two groups was performed using the unpaired Student’s *t*-test (for normally distributed data) or the Wilcoxon rank-sum test (for non-normally distributed data). Comparison among more than two groups was carried out using one-way ANOVA (for normally distributed variables) or the Kruskal–Wallis test (for non-normal data). Paired comparisons of data over two time points were performed using the paired Student’s *t*-test (for normal data) or the Wilcoxon signed-rank test (for non-normal data). These tests were applied to both primary and secondary clinical endpoints, depending on the distribution and type of the variable.

The significance level was set at *p* < 0.05. All statistical analyses were performed using Stata v.13.0 for Macintosh.

## 3. Results

**Figure 1:**
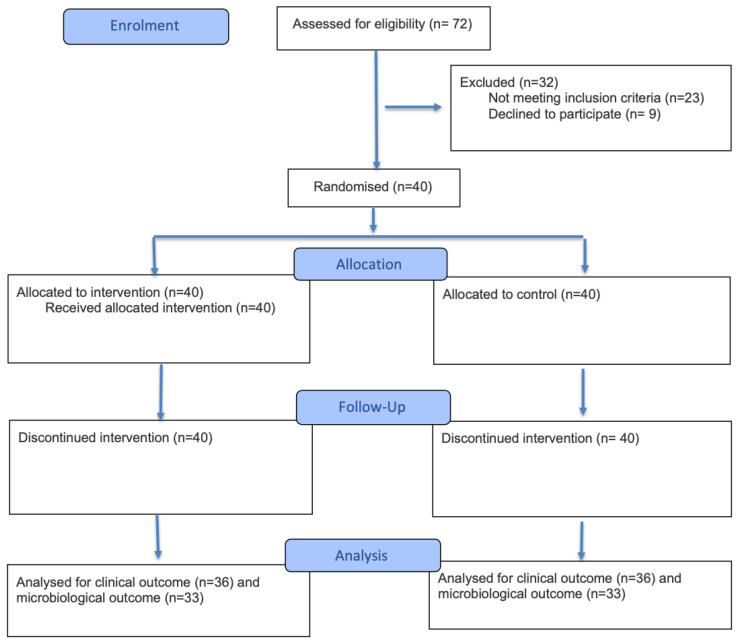
Consort flow diagram of the progress through the phases of a randomized trial.

An initial screening was conducted on 72 patients (see Figure 1). At the end of the screening phase, a total of 40 patients who met the inclusion criteria were enrolled in the study. The patients enrolled presented a diagnosis of stage II or III periodontitis (grades A to C). Periodontal defects presented as stage II in twenty-five cases and stage III in fifteen cases, in addition to grade A in twelve cases, grade B in twenty-six cases, and grade C in two cases. Among the 40 patients who agreed to take part in the treatment phase, 2 patients did not attend the follow-up visits at T45d (follow-up 45 days) and T90d (follow-up 90 days), and one patient did not attend the follow-up visit at T90d.

### 3.1. Clinical Outcomes

At Tbase, there were no significant differences in terms of clinical parameters between the control group and the test groups.

Regarding PPD (see Table 1), in the control group, a mean reduction in PPD of 1.4 mm at T45d and 1.1 mm at T90d, compared to Tbase, was observed. For the group HBX 1T, a mean PPD reduction of 1.5 mm at T45d and 1.6 mm at T90d was recorded. For the group HBX 3T, the mean PPD decreased by 1.7 mm at T45d and 1.6 mm at T90d. Statistical analysis revealed no statistically significant differences in mean PPD values between treatment groups at Tbase and T45d. However, a statistically significant difference was observed between groups at T90d (*p* = 0.04) and for the interval between Tbase and T90d (*p* = 0.04). All treatment groups showed a statistically significant reduction (*p* < 0.05) in mean PPD between different time intervals.

Regarding BOP (see Table 2), in the site treated with the desiccant gel in a single session, a reduction in BOP of 42% at T45d and 29% at T90d was observed compared to Tbase. In the sites treated with the gel in all three sessions, the reduction was 30% at T45d and 29% at T90d. Statistical analysis showed no statistically significant differences in mean BOP between groups at Tbase, T45d, and T90d. However, all treatment groups showed a statistically significant reduction (*p* < 0.05) in mean BOP between different time intervals.

Regarding PI (see Table 3), significant reductions (*p* < 0.05) were observed in all treatment groups between Tbase and T45d or T90d. At T45d, the mean PI decreased by 0.8 in the control group, 0.5 in the HBX 1T, and 0.6 in the HBX 3T, compared to Tbase. At T90d, the reductions were 0.8 (control group), 0.5 (HBX 1T), and 0.7 (HBX 3T), respectively. Statistical analysis showed no statistically significant differences in mean PI between groups at Tbase, T45d, and T90d. However, all treatment groups showed a statistically significant reduction (*p* < 0.05) in mean PI between different time intervals.

Regarding REC (see Table 4), at T45d, an increase in mean REC of 0.6 mm was observed in the control group, 0.5 mm in the HBX 1T, and 0.5 mm in the HBX 3T, compared to Tbase. At T90d, the mean increase in REC was 0.6 mm in the control group, 0.4 mm in the HBX 1T, and 0.5 mm in the HBX 3T. All treatment groups showed an increase in REC from Tbase to T45d or T90d. Statistical analysis showed no statistically significant differences in mean REC between groups at Tbase, T45d, and T90d. However, all treatment groups showed a statistically significant reduction (*p* < 0.05) in mean REC between different time intervals.

Regarding CAL (see Table 5), statistical analysis showed no significant differences in CAL between treatment groups at Tbase, T45d, or T90d. However, a statistically significant difference (*p* = 0.02) was observed between groups for variations from Tbase to T90d. Each group showed a statistically significant improvement (*p* < 0.05) in mean CAL from Tbase to T90d.

### 3.2. Microbiological Outcomes

**Figure 2:**
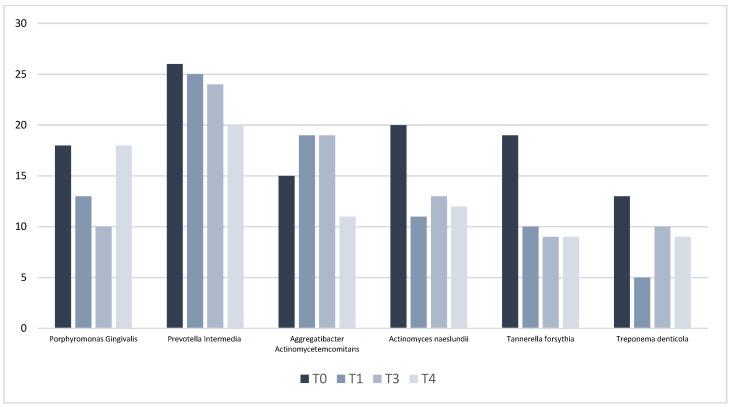
Microbiological analysis of group HBX 1T.

**Figure 3:**
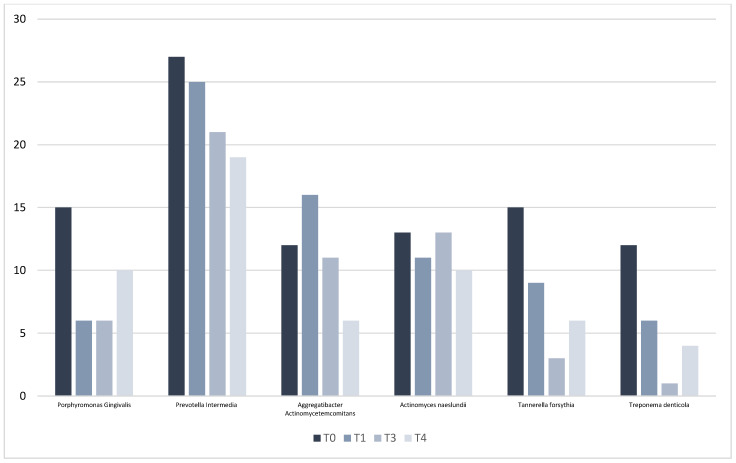
Microbiological analysis of group HBX 3T.

The following section analyzes microbiological data obtained from subgingival plaque samples collected from the study sites. During microbiological data processing, the quantification of uncolonized pockets, control pockets colonized, HBX 1T pockets colonized, and HBX 3T pockets colonized by the target periodontopathogen was performed and can be visible in Figure 2 and Figure 3. Microbiological evaluation confirmed greater reductions in *Aggregatibacter actinomycetemcomitans*, *Porphyromonas gingivalis*, *Prevotella intermedia*, *Tannerella forsythia*, and *Treponema denticola* in HBX 3T at T45d and T90d, while *Actinomyces naeslundii* reduction was greater in the control group.

## 4. Discussion

The primary objective of non-surgical periodontal therapy is to reduce the bacterial load both above and below the gumline, thereby preventing the progression of chronic periodontitis [6]. As previously discussed, disease onset and progression are driven by two key mechanisms: bacterial biofilm accumulation and the host inflammatory response. Although scaling and root planing (SRP) remain fundamental, evidence in the literature suggests that SRP alone may not be sufficiently effective in halting disease progression [31]. In this context, the use of adjunctive strategies [32], such as the topical desiccant HybenX gel, has emerged as a promising option.

HYBENX primarily acts on the biofilm extracellular matrix (EPS) through a contact desiccation and denaturation mechanism, causing dehydration and structural weakening of the biofilm that facilitates its mechanical removal with scaling and ultrasonic debridement; thus, its principal role is as a facilitator of biofilm disruption rather than as a deeply penetrating bactericidal agent [23,30]. By contrast, other adjunctive therapies—such as lasers, photodynamic therapy (PDT), ozone therapy, antiseptic gels, or local/systemic antibiotics [33,34,35,36]—exert effects that are more directly antimicrobial via oxidative, photothermal, or chemical mechanisms and, in some cases, can reduce microbial loads in deeper biofilm layers or tissue niches where desiccation alone may be insufficient. Each modality, however, has intrinsic limitations (e.g., variable penetration into mature EPS, need for specific devices or operator skill, issues of dose standardization, potential tissue effects, or risk of antimicrobial resistance), so HYBENX should be viewed as a complementary, locally acting tool that can improve the efficacy of mechanical debridement, while definitive conclusions about comparative effectiveness require well-designed, adequately powered trials with quantitative microbiological endpoints.

However, only a few studies assessed their efficacy in conjunction with SRP in reducing biofilm and inflammatory mediators [37,38]. In this study, clinical outcomes of SRP alone were compared with SRP + HBX, applied in the dental pocket either once (HBX 1T) or three times (HBX 3T), to determine whether HBX offers additional benefits.

Our study revealed consistent improvements in PPD, BOP, PI, and CAL across all treatment groups compared to baseline, with an increase in REC, as an expected consequence of PPD reduction.

All treatment groups showed a statistically significant reduction (*p* < 0.05) in mean PPD between different time intervals. Clinically speaking, we can therefore deduce that one application and three applications do not change the situation. These findings are comparable with those of Isola et al. (2018), who reported significantly greater PPD reduction in the SRP + HBX group at 12 months (SRP: 2.23 ± 0.31 mm vs. SRP + desiccant: 3.25 ± 0.57 mm, *p* < 0.001) [2]. Iorio-Siciliano et al. [39] found improved outcomes using a local application of an amino acid buffered sodium hypochlorite (NaOCl)-amino acid gel in addition to MINST compared to MINST alone (the number of sites with PD ≥ 5 mm and BOP (+) decreased statistically significantly from 85.3 to 2.2% in the test group and from 81.6 to 7.3% in the control group, respectively, *p* = 0.001). Ramanauskaite et al. [40] demonstrated improvement in clinical parameters with an SRP + NaOCl-hyaluronic acid protocol (PD reduction: 2.9 ± 0.4 vs. 1.8 ± 0.6 mm; BOP decreased from 81.8 ± 16.2% to 48.9 ± 14.5% in control and from 83.2 ± 15.5% to 17.6 ± 11.5% in test (*p* < 0.001) groups). Conversely, Lombardo et al. [23] and Soanca et al. [41] reported no significant PPD differences with the addition of HybenX gel to SRP. A meta-analysis by Pardo et al. [16] supported the benefit of phenolic/sulfonated agents at 3 and 6 months, though it called for further randomized trials due to heterogeneity.

A reduction in BOP was observed compared to Tbase. In the sites treated with the gel in all three sessions, the total reduction was 30%. All treatment groups showed a statistically significant reduction (*p* < 0.05) in mean BOP between different time intervals, a result that aligns with the PPD index. Khalil et al. [22] reported significantly greater BOP reduction with SRP + HBX at 3 and 6 months (*p* < 0.001). Isola et al. [2] (64% of BOP reduction in the test group vs. 35% for the control group at 12 months) and Lombardo et al. [23] had similar findings. Furthermore, it was also reported that the use of HybenX gel in periodontal abscess can improve inflammatory indices [42]. Radulescu et al. [43] found significant BOP differences in favor of SPT + NaOCl at 12 months, while Iorio-Siciliano et al. [39] noted greater BOP reduction in the test group, with a reduction from 85.3% to 2.2% vs. 81.6% to 7.3% for the control group at 6 months (*p* = 0.001).

As for the plaque index, the results of the single treatment and the three treatments are similar, and the statistical analysis showed no statistically significant differences in mean PI between groups. However, all treatment groups showed a statistically significant reduction (*p* < 0.05) in mean PI between different time intervals. These results agree with Isola et al., who found no significant PI differences (17.1% vs. 22.8%, *p* = 0.071) [2], as well as with Khalil et al. [22]. Lombardo et al. observed lower PI in HBX-treated sites [23]. Zafar et al. reported significantly enhanced plaque removal with SRP + HBX (*p* < 0.001), though calculus removal was unchanged [43,44]. The hydration-disrupting action of HBX supports manual or ultrasonic removal [45]; in our study, repeated SRP may have equalized PI outcomes across groups.

All treatment groups showed an increase in REC from Tbase to T45d or T90d. Statistical analysis showed no statistically significant differences in mean REC between groups at Tbase, T45d, and T90d. However, all treatment groups showed a statistically significant reduction (*p* < 0.05) in mean REC between different time intervals. These outcomes indicate that HBX does not harm the periodontium, consistent with findings of Lombardo et al., Isola et al., and Khalil et al. [2,22,23].

Statistical analysis showed no significant differences in CAL between treatment groups at different follow-ups. Each group showed a statistically significant improvement (*p* < 0.05) in mean CAL from Tbase to T90d. The improvement of CAL in HBX groups is corroborated by the results of the studies of Isola et al., Khalil et al. [2,22], and Ramanauskaite et al. [40], who reported a 2.6 ± 0.5 mm gain in the test group vs. 1.6 ± 0.6 mm in the control group (*p* < 0.001).

Microbiological analysis revealed that HBX 3T led to more significant reductions in periodontopathogenic bacteria such as *Aggregatibacter actinomycetemcomitans*, *Porphyromonas gingivalis*, *Prevotella intermedia*, *Tannerella forsythia*, and *Treponema denticola*, whereas the control group showed a greater decrease in *Actinomyces naeslundii*. This latter bacterium is typically found in the human mouth and is generally regarded as a benign commensal organism. These findings are consistent with those of other studies [2,23]. Although traditional NSPT temporarily lowers bacterial levels, they tend to increase again after three months. In contrast, sites treated with repeated HBX applications demonstrated more sustained anaerobic suppression, supporting the idea that multiple HBX treatments can improve long-term management of pathogenic biofilm through matrix disruption and mechanical facilitation. Besides its antimicrobial effects, HBX serves as a unique adjunctive therapy, not only for its antiseptic properties but also as a biofilm-disrupting agent, used before mechanical debridement. This application takes advantage of HBX’s strong affinity for water to effectively absorb the biofilm’s aqueous component, leading to its denaturation and subsequent breakdown; a critical process, given the well-documented resistance of mature biofilms to both mechanical and chemical removal.

The outcomes of other randomized clinical trials highlight a strong limitation for the present study, which is the lack of long-term follow-up at 6 and 12 months, which would have allowed a more precise evaluation of the effects of the treatment with HybenX gel + US-SRP, as well as the stability of the outcomes achieved at 3 months. Another possible limitation is related to the presence of three treatment groups, which provides potential biological interference, that is represented by the bias of adjacent sites. Furthermore, it is difficult to ensure that the patient has maintained the same level of oral hygiene across the different treated sites, as they may have been psychologically influenced by knowing that different treatments were applied in different areas of the mouth. It should be noted that, from a microbiological point of view, a qualitative investigation was carried out (presence or absence of the microorganism’s genome). In the future, it could be hypothesized to perform a quantitative analysis, using real-time PCR or digital PCR, at the sites that remain positive even after treatment, to assess the reduction in the bacterial load at different follow-up intervals.

## 5. Conclusions

This randomized clinical trial investigated different treatment modalities, including conventional non-surgical periodontal therapy, and the application of a desiccating plaque agent (HybenX gel) in conjunction with full-mouth ultrasonic debridement as well as scaling and root planing, resulting in significant clinical improvements for all groups. Overall, positive results showed greater reductions in PPD and CAL in the HBX-treated groups compared to the control group. Notably, a single application of HBX yielded clinical results comparable to three applications; nevertheless, repeated applications achieved a more substantial bacterial reduction. These findings suggest that the inclusion of HBX in periodontal treatment protocols may enhance clinical outcomes and microbial control, potentially offering an effective adjunct to conventional periodontal therapy. These findings are preliminary and require confirmation in larger, well-powered trials with quantitative microbiological outcomes and longer follow-up before firm clinical recommendations can be made.

## Figures and Tables

**Table 1:** PPD (Probing Pocket Depth).

PPD (mm)	T0	T3	T4	Δ(T0–T3)	*p* Value	Δ(T3-T4)	*p* Value	Δ(T0–T4)	*p* Value
Total	5.2 ± 0.43	3.7 ± 0.90	3.8 ± 0.94	1.5 ± 0.85	0.001 *	0.1 ± 0.58	0.24	1.4 ± 0.90	0.001 *
Control	5.2 ± 0.42	3.9 ± 0.91	4.1 ± 1.0	1.4 ± 0.82	0.001 *	0.2 ± 0.54	0.02 *	1.1 ± 0.88	0.001 *
HBX1T	5.2 ± 0.36	3.7 ± 0.85	3.7 ± 0.9	1.5 ± 0.83	0.001 *	0.1 ± 0.57	0.3	1.6 ± 0.93	0.001 *
HBX3T	5.3 ± 0.49	3.6 ± 0.92	3.7 ± 0.8	1.7 ± 0.88	0.001 *	0.1 ± 0.58	0.7	1.6 ± 0.81	0.001 *
*p* Value	0.61	0.36	0.04 *	0.28		0.04 *		0.04 *	

Outcomes are expressed in mm as mean ± sd (standard deviation); * = statistically significant difference between groups or time intervals.

**Table 2:** BOP (Bleeding on Probing).

BOP (%)	T0	T3	T4	Δ(T0–T3)	*p* Value	Δ(T3-T4)	*p* Value	Δ(T0–T4)	*p* Value
Total	73 ± 0.44	38 ± 0.51	43 ± 0.50	34 ± 0.61	0.002 *	4 ± 0.56	0.7	30 ± 0.63	0.001 *
Control	69 ± 0.46	39 ± 0.49	43 ± 0.5	28 ± 0.61	0.002 *	3 ± 0.51	0.7	26 ± 0.61	0.001 *
HBX1T	77 ± 0.42	33 ± 0.47	46 ± 0.5	42 ± 0.55	0.002 *	11 ± 0.62	0.28	29 ± 0.62	0.007 *
HBX3T	72 ± 0.45	44 ± 0.55	43 ± 0.5	30 ± 0.66	0.01 *	3 ± 0.55	0.7	29 ± 0.66	0.01 *
*p* Value	0.74	0.72	0.96	0.61		0.56		0.96	

Outcomes are expressed in % as mean ± sd (standard deviation); * = statistically significant difference between groups or time intervals.

**Table 3:** PI (Plaque Index).

PI	T0	T3	T4	Δ(T0–T3)	*p* Value	Δ(T3-T4)	*p* Value	Δ(T0–T4)	*p* Value
Total	1.3 ± 1.17	0.7 ± 0.88	0.6 ± 0.91	0.6 ± 1.27	0.002 *	0.0 ± 1.03	0.9	0.7 ± 1.36	0.001 *
Control	1.5 ± 1.2	0.7 ± 0.97	0.6 ± 0.96	0.8 ± 1.41	0.002 *	0.1 ± 1.31	0.9	0.8 ± 1.62	0.001 *
HBX1T	1 ± 1.13	0.5 ± 0.77	0.5 ± 0.85	0.5 ± 1.06	0.006 *	0.03 ± 0.73	0.78	0.5 ± 1.14	0.003 *
HBX3T	1.4 ± 1.15	0.8 ± 0.87	0.8 ± 0.93	0.6 ± 1.32	0.007 *	0.1 ± 0.93	0.55	0.7 ± 1.26	0.002 *
*p* Value	0.09	0.29	0.38	0.65		0.96		0.53	

Dimensionless outcomes are expressed as mean ± sd (standard deviation); * = statistically significant difference between groups or time intervals.

**Table 4:** REC (Gingival Recession).

REC (mm)	T0	T3	T4	Δ(T0–T3)	*p* Value	Δ(T3-T4)	*p* Value	Δ(T0–T4)	*p* Value
Total	0.8 ± 1.04	1.4 ± 1.17	1.4 ± 1.18	0.6 ± 0.52	0.001 *	0.3 ± 0.87	0.2	0.5 ± 0.63	0.001 *
Control	0.8 ± 1.01	1.5 ± 1.13	1.5 ± 1.17	0.6 ± 0.56	0.001 *	0.0 ± 0.41	0.2	0.6 ± 0.48	0.001 *
HBX1T	0.8 ± 0.97	1.3 ± 1.12	1.2 ± 1.02	0.5 ± 0.52	0.006 *	0.1 ± 0.32	0.8	0.4 ± 0.55	0.003 *
HBX3T	0.8 ± 1.14	1.4 ± 1.25	1.4 ± 1.32	0.5 ± 1.32	0.001 *	0.1 ± 0.37	0.08	0.5 ± 0.58	0.001 *
*p* Value	0.72	0.69	0.78	0.32		0.56		0.62	

Outcomes are expressed in mm as mean ± sd (standard deviation); * = statistically significant difference between groups or time intervals.

**Table 5:** CAL (Clinical Attachment Level).

CAL (mm)	T0	T3	T4	Δ(T0–T3)	*p* Value	Δ(T3-T4)	*p* Value	Δ(T0–T4)	*p* Value
Total	6.0 ± 1.18	5.1 ± 1.77	5.2 ± 1.72	0.9 ± 0.93	0.001 *	0.0 ± 0.64	0.06	0.9 ± 0.96	0.001 *
Control	6.1 ± 1.23	5.3 ± 1.87	5.6 ± 1.84	0.7 ± 0.94	0.07	0.2 ± 0.48	0.45	0.5 ± 1.02	0.03 *
HBX1T	5.8 ± 1.39	4.9 ± 1.65	4.7 ± 1.56	1.1 ± 1.20	0.001 *	0.2 ± 0.74	0.22	1.3 ± 1.17	0.001 *
HBX3T	6.1 ± 1.29	5.0 ± 1.85	5.1 ± 1.76	1.1 ± 0.88	0.98	0.0 ± 0.61	0.05 *	1.1 ± 0.82	0.001 *
*p* Value	0.61	0.59	0.13	0.8		0.78		0.02*	

Outcomes are expressed in mm as mean ± sd (standard deviation); * = statistically significant difference between groups or time intervals.

## Data Availability

The original contributions presented in this study are included in the article. Further inquiries can be directed to the corresponding authors.
